# Improved Histone Deacetylase Inhibitors as Therapeutics for the Neurodegenerative Disease Friedreich's Ataxia: A New Synthetic Route

**DOI:** 10.3390/ph4121578

**Published:** 2011-12-14

**Authors:** Chunping Xu, Elisabetta Soragni, Vincent Jacques, James R. Rusche, Joel M. Gottesfeld

**Affiliations:** 1 Department of Molecular Biology, The Scripps Research Institute, 10550 N. Torreys Pines Road, La Jolla, CA 92037, USA; E-Mails: chpxu@scripps.edu (C.X.); soragni@scripps.edu (E.S.); 2 Repligen Corporation, 41 Seyon Street, Waltham, MA 02453, USA; E-Mails: vjacques@repligen.com (V.J.); jrusche@repligen.com (J.R.R.)

**Keywords:** HDAC inhibitor, Friedreich's ataxia, click chemistry

## Abstract

Friedreich's ataxia (FRDA) is caused by transcriptional repression of the nuclear *FXN* gene encoding the essential mitochondrial protein frataxin. Based on the hypothesis that the acetylation state of the histone proteins is responsible for gene silencing in FRDA, previous work in our lab identified a first generation of HDAC inhibitors (pimelic *o*-aminobenzamides), which increase *FXN* mRNA in lymphocytes from FRDA patients. Importantly, these compounds also function in a FRDA mouse model to increase *FXN* mRNA levels in the brain and heart. While the first generation of HDAC inhibitors hold promise as potential therapeutics for FRDA, they have two potential problems: less than optimal brain penetration and metabolic instability in acidic conditions. Extensive optimization focusing on modifying the left benzene ring, linker and the right benzene ring lead to a novel class of HDAC inhibitors that have optimized pharmacological properties (increased brain penetration and acid stability) compared to the previous HDAC inhibitors. This article will describe the chemical synthesis and pharmacological properties of these new HDAC inhibitors.

## 1. Introduction

**Figure 1:**
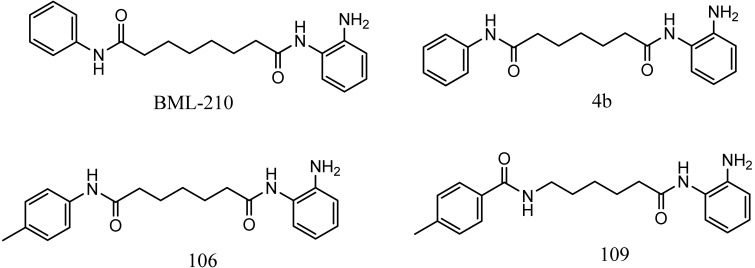
Structures of HDAC inhibitors *

Friedreich's ataxia (FRDA) is caused by transcriptional repression of the nuclear *FXN* gene encoding the essential mitochondrial protein frataxin [1-3]. Currently there is no effective therapy for FRDA that addresses the cause of the disease. Unlike many triplet-repeat diseases (e.g., the polyglutamine expansion diseases), expanded GAA**•**TTC triplets in *FXN* are in an intron and do not alter the amino acid sequence of frataxin protein; thus, gene activation would be of therapeutic benefit. Based on the hypothesis that chromatin structure and the histone modification state of the *FXN* gene are responsible for gene repression on alleles containing expanded GAA•TTC repeats, our lab found that a commercially available histone deacetylase inhibitor (BML-210, Figure 1) and derivatives **4b**, **106** and **109** that we synthesized (Figure 1) relieve repression of the *FXN* gene in lymphoid cell lines derived from FRDA patients, in primary lymphocytes from donor FRDA patient blood [4-6], and in mouse models [7-9]. Recently, these compounds have also shown efficacy in FRDA patient iPSC-derived neuronal cells [10].

**Figure 2:**
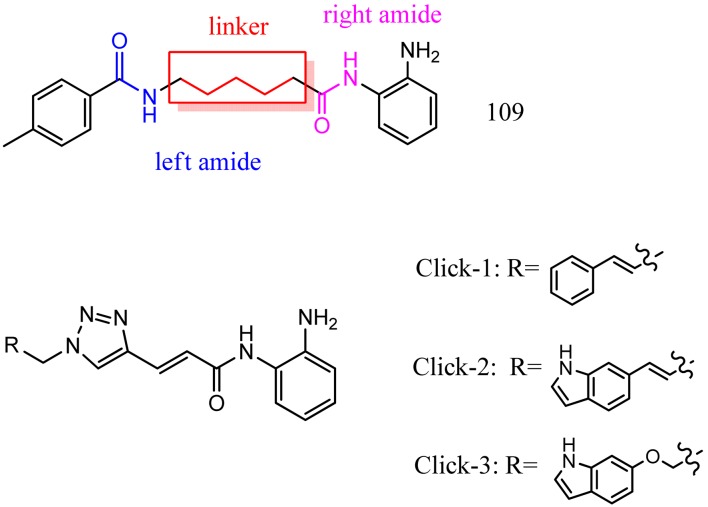
Structures of **109** and click compounds.

Optimization of our original series of compounds, exemplified by **4b** [4] and **106** [7] lead to the development of HDACi **109** [8,9]. This compound has been subjected to full preclinical development and will soon enter human clinical trials for FRDA. While **109** holds promise as a FRDA therapeutic, this molecule has two potential problems: less than optimal brain penetration [11] and cyclization to a benzimidazole *in vivo*. We have identified two structural features that solve these problems: replacement of the “left” amide with an ether, olefin or ketone to improve brain penetration and introduction of an unsaturated linkage adjacent to the “right” amide to prevent formation of a benzimidazole metabolic byproduct. Based on these results, we devised and synthesized a new lead compound click-1 (Figure 2) using Cu(I)-catalyzed click chemistry [12]. Structure-activity relationship at the left side (cap) of the molecule was evaluated by replacement of the left benzene ring with an indole ring, which generated derivative click-2. The left double bond on click-2 was replaced with an ether to give another derivative click-3 (Figure 2). Activity against recombinant HDACs (HDAC1, 2 and 3), activity in restoring *FXN* transcription in patient cell lines, blood brain barrier penetration and acid stability have been assayed for each of these derivatives.

## 2. Results and Discussion

**Scheme 1:**
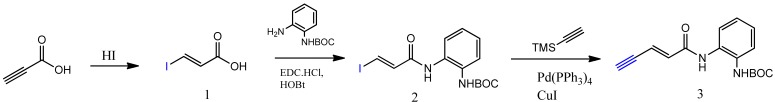
Synthesis of common intermediate 3.

The synthesis of compounds click-1, -2, and -3 relies on the formation of a triazole ring by coupling of a common enyne intermediate with various azides using a Cu(I) catalyst. Hydrogen iodide addition to propiolic acid generated acrylic iodide **1**, which was subjected to standard amide coupling with *N*-Boc-1,2-phenylenediamine to give compound **2**. Sonogashira coupling of vinyl iodide **2** with trimethylsilyl acetylene lead to the common intermediate enyne **3** for the following click chemistry (Scheme 1).

**Scheme 2:**
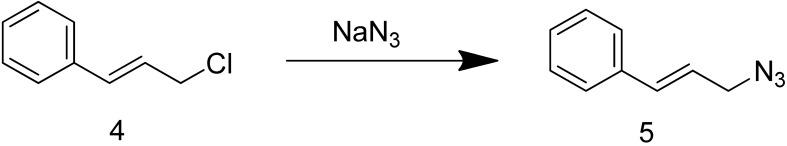
Synthesis of azide 5.

Cinnamyl azide **5** was prepared by treating commercially available cinnamyl chloride **4** with sodium azide (Scheme 2).

Both 6-(2-azidoethoxy)-1*H*-indole (**8**) and (*E*)-*N*-(*t*-butoxycarbonyl)-6-(3-azidoprop-1-en-1-yl)-1*H*-indole (**16**) were synthesized by Aberjona Laboratories (Beverly, MA, USA) through the following synthetic routes.

**Scheme 3:**
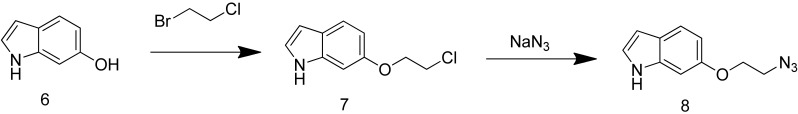
Synthesis of azide **8**.

6-(2-azidoethoxy)-1*H*-indole (**8**) was obtained through reaction of sodium azide with the corresponding chloride **7**, which was prepared by substitution reaction of 6-hydroxy-1*H*-indole with 1-bromo-2-chloroethane (Scheme 3).

**Scheme 4:**
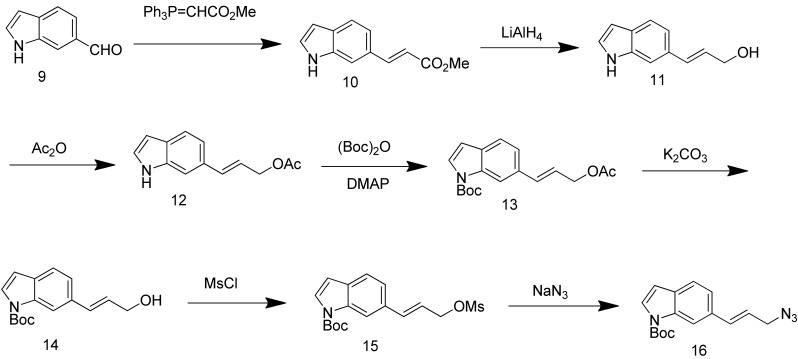
Synthesis of azide **16**.

Synthesis of (*E*)-*N*-(*t*-butoxycarbonyl)-6-(3-azidoprop-1-en-1-yl)-1*H*-indole (**16**) started with commercially available compound 1*H*-indole-6-carbaldehyde (**9**). Wittig olefination of aldehyde **9** afforded compound **10**. Reduction of the ester **10**, followed by acetylation of the alcohol **11** lead to compound **12**. Boc protection of the indole amine, removal of acetyl group with potassium carbonate, followed by mesylation of the free alcohol **14** generated compound **15**, which was reacted with sodium azide to give azide **16** (Scheme 4).

**Scheme 5:**
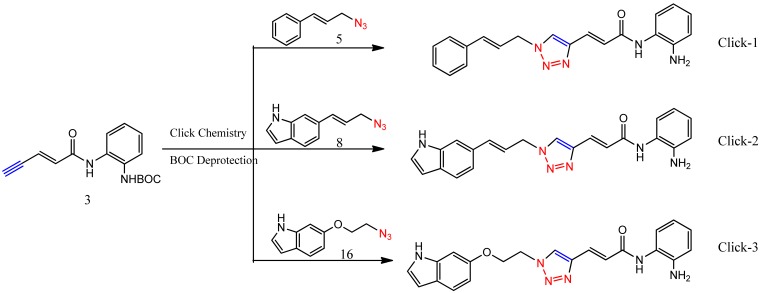
Synthesis of click compounds.

All three azides **5**, **8** and **16** were subjected to click chemistry with common intermediate **3** to give the corresponding triazole products click-1, click-2 and click-3 (Scheme 5). Each compound was characterized by ^1^H-, ^13^C-NMR and mass spectrometry. In vitro HDAC assays, in-cell deacetylase (DAC) assays, and cellular assays for effects on *FXN* transcription were carried out for all three click compounds, and the results compared to those for the original lead compound **109**. Compound click-3 displayed poor stability; hence, studies with this compound were not pursued further.

*HDAC assays*: Previous studies have established that the pimelic *o*-aminobenzamides are potent inhibitors of class I HDAC enzymes [6]. Therefore, the inhibitory activity of compounds click-1 and click-2 against the recombinant class I HDACs 1-3 was determined (Table 1). By measuring IC_50_ values, we find that these compounds retain full activity as inhibitors of these enzymes, compared to HDACi **109**. In fact, both click compounds exhibit lower IC_50_ values against these three enzymes than **109**. Notably, the click compounds are more potent against HDACs 1 and 2 than **109**. This increased activity against HDACs 1 and 2 reduces the selectivity of the click compounds for HDAC3, where compound **109** has a 4.3-fold selectivity for HDAC3 over HDAC1, while click-1 and -2 are only 2.4- and 1.2-fold selective for HDAC3 over HDAC1, respectively. In-cell efficacy of these compounds, as measured in the DAC assay (see Experimental section), is also not significantly different from **109**. It should be also pointed out that both click-1 and click-2 have comparable inhibitory activity against recombinant HDACs compared to the well-studied benzamide MS-275 (IC_50_s: HDAC1 = 0.036 μM; HDAC2 = 0.219 μM; HDAC3 = 0.114 μM and DAC = 1.9 μM); however, MS-275 has cytotoxicity issue and does not penetrate the central nervous system [15].

*Cytotoxicity assays*: Cytotoxicity was determined using an antiproliferation assay with Hct116 cells. MS-275, being cytotoxic, gave an IC_50_ of 1 μM, while click-1 and click-2 had IC_50_s = 23 and 27 μM respectively. Comparing these IC_50_ values for blocking cell proliferation to the in-cell DAC assay values (Table 1) yields ratios of 0.53 for MS-275 (cytotoxicity/DAC), 4.1 for click-1 and 5.3 for click-2. Thus, our click compounds demonstrate a more favorable therapeutic index for a chronic disease compared to MS-275.

*Frataxin mRNA assays*: Through target identification, our lab has recently demonstrated that HDAC3 plays a key role in *FXN* gene transcription [5]. In general, the activity of a compound against HDAC3 is related to its ability to increase *FXN* gene transcription [4]. Compounds click-1 and -2 were assayed for their ability to increase transcription of the *FXN* gene in primary lymphocytes from a FRDA patient, as previously described [4,5].

**Figure 3:**
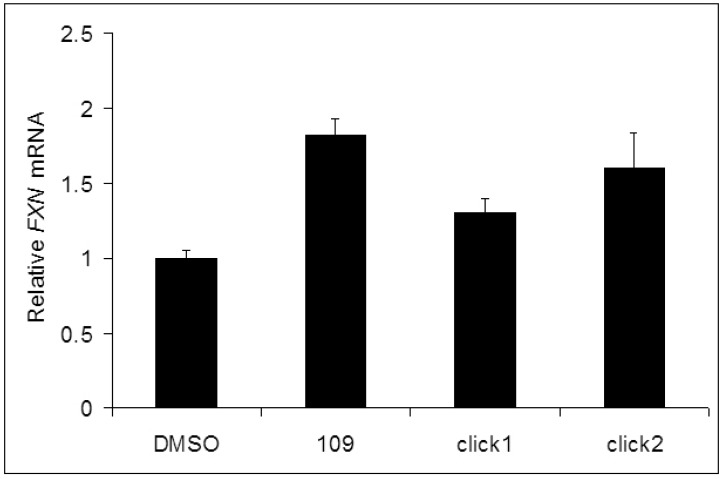
Click-derived HDAC inhibitors induce frataxin gene expression in primary lymphocytes isolated from FRDA patient donor blood samples, compared to a standard pimelic *o*-aminobenzamide (**109**). Concentration of each compound is 10 μM.

Quantitative RT-PCR was used to estimate *FXN* mRNA levels after treatment with 10 μM compound for 48 h, and values were normalized to the level of *FXN* mRNA observed for a DMSO vehicle control. Importantly, compounds click-1 and click-2 are highly active in promoting transcription of the silenced *FXN* gene in primary lymphocytes isolated from Friedreich's ataxia patient donor blood, pointing to the therapeutic potential of these compounds (Figure 3).

*Brain penetration and acid stability*: Since HDACi **109** suffers from poor brain penetration (∼10–15% of plasma levels) and from formation of a benzimidazole under acid conditions, we monitored these two properties for click-1 and click-2, and compared the results with those obtained for HDACi **109**. The half-life of each compound in acidic conditions was used to quantify their relative stability. At pH = 2 and 50 °C, t_1/2_ = 6 h for **109**, t_1/2_ = 33 h for click-1 and t_1/2_ = 24 h for click-2, representing improvements of 5.5- and 4-fold respectively relative to HDACi **109**. These results demonstrate that placing a double bond next to the right amide bond improves stability in acidic conditions. Blood brain barrier penetration was measured in the rat for compound **109** and click-1. Brain penetration (as measured by brain/plasma ratio at t_max_) was determined to be 0.15 for **109** and 0.33 for click-1 (Cmax: 800 ng/g in the brain at Tmax = 5 min post-dose (5 mg/kg i.v. rat)), representing a significant increase in brain penetration for click-1. Click-2 was tested in mouse only, where brain penetration was limited with a brain/plasma of 0.03, as can be expected because of the indole cap (relatively hydrophilic and capable of intermolecular H-bonding).

## 3. Experimental

### 3.1. General

Reagents were purchased at the highest commercial quality and used without further purification, unless otherwise stated. NMR spectra were recorded on Bruker DRX-500, Varian Inova-400 and Varian Mercury-300 instruments and calibrated using residual undeuterated solvent as an internal reference (CHCl_3_ @ 7.24 ppm ^1^H-NMR, 77.0 ppm ^13^C-NMR, CD_3_OD @ 3.35 ppm ^1^H-NMR, 49.3 ppm ^13^C-NMR). High resolution mass spectra (HRMS) were recorded at the Center of Mass Spectrometry, the Scripps Research Institute.

### 3.2. (E)-Tert-butyl (2-(3-Iodoacrylamido)phenyl)carbamate (**2**)


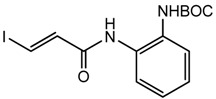


(*E*)-3-Iodo-2-propenoic acid (0.40 g, 2.02 mmol) was dissolved in anhydrous dichloromethane (8 mL) under argon protection. Oxalyl chloride (0.34 mL, 3.96 mmol) was added dropwise to the above solution, one drop of DMF was added to catalyze the reaction, and the reaction was stirred at room temperature for 2 h. After removal of the solvent, the residue was used directly in the next step. Anhydrous dichloromethane (10 mL) was added to the above acid chloride, and N-Boc phenylenediamine (0.35 g, 1.68 mmol) was added to the solution, followed by diisopropylethylamine (0.42 mL, 2.42 mmol). The reaction was kept at room temperature for 1 h. Removal of the solvent followed by column chromatography gives 0.51 g of product **2**. Yield: 78%; ^1^H-NMR (300 MHz, CD_3_OD) (ppm): 8.53 (1H), 8.06 (d, 1H, *^3^J* = 15Hz), 6.96 (d, 1H, ^3^ *J* = 15Hz), 6.92–7.50 (4H), 1.52 (s, 9H); ^13^C-NMR (75 MHz, CD_3_OD) (ppm): 165.7, 137.2, 135.0, 129.7, 128.2, 127.4, 126.6, 125.7, 99.3, 77.6, 27.6.

### 3.3. (E)-Tert-butyl (2-(Pent-2-en-4-ynamido)phenyl)carbamate (**3**)


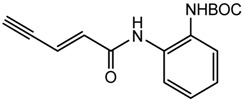


Iodo propenamide **2** (0.19 g, 0.49 mmol) was dissolved in DMF/Et_3_N (4:1, 2 mL), Pd(PPh_3_)_2_Cl_2_ (17.3 mg, 0.0245 mmol) and copper iodide (9.4 mg, 0.049 mmol) were added to the solution and the mixture was degassed for 5 minutes. Trimethylsilylacetylene (0.13 mL, 0.91 mmol) was added to the above solution under argon protection. The whole reaction was kept at room temperature for 1 h. The mixture was then poured into sat. (NH_4_)_2_SO_4_ (50 mL) and extracted with ethyl acetate (50 mL × 3). The combined organic layers were washed with brine, dried with sodium sulfate, filtered, and concentrated. The residue was subjected to flash column chromatography to yield 0.15 g trimethylsilyl enyne product, yield: 86%. The trimethylsilyl enyne product (0.15 g, 0.42 mmol) was redissolved in THF/Methanol (1:1, 2 mL), cesium fluoride (76 mg, 0.50 mmol, 1.2 eq.) was then added and the reaction was monitored by TLC analysis. The product is slightly more polar. Upon the disappearance of the reactant, the reaction mixture was concentrated and purified by column chromatography to yield the enyne product **3** (0.11 g, 95%); ^1^H-NMR (300 MHz, CDCl_3_) (ppm): 8.59 (1H), 6.97–7.47 (4H), 6.78 (dd, 1H, *^3^J*= 15.3 Hz, *^4^ J*= 2.4 Hz), 6.44 (d, 1H, *^3^J* = 15.3 Hz), 3.32 (s, 1H), 1.52 (s, 9H); ^13^C-NMR (75 MHz, CDCl_3_) (ppm): 166.2, 135.0, 129.7, 126.6, 125.7, 124.6, 121.8, 81.5, 80.8, 77.6, 28.5.

### 3.4. 6-(2-Azidoethoxy)-1H-indole (**8**)


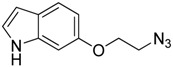


Sodium azide (416 mg, 2 eq.) was added to 6-(2-chloroethoxy)-1H-indole (626 mg, 3.2 mmol) in DMSO (10 mL) at room temperature. The mixture was heated for 8 h at 80 °C. After the mixture was cooled to room temperature it was diluted with CH_2_Cl_2_ and washed twice with water. The organic layer was dried over Na_2_SO_4_, filtered, and concentrated. The residue was purified by reversed phase preparative LC to afford compound **8**, (425 mg, 66%); ^1^H-NMR (300 MHz, CDCl_3_) (ppm): 8.04 (br s, 1H), 7.54 (d, *^3^J* = 8.7 Hz), 7.12 (m, 1H), 6.91 (br s, 1H), 6.83 (dd, *^3^J* = 8.7 Hz and *^4^J* = 2.1 Hz, 1H), 6.50 (m, 1H), 4.19 (t, *^3^J* = 4.8 Hz, 2H), 3.61 (t, *^3^J* = 4.8 Hz, 2H).

### 3.5. (E)-Tert-butyl 6-(3-((Methylsulfonyl)oxy)-prop-1-en-1-yl)-1H-indole-1-carboxylate (**15**)


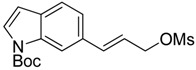


The mesylate **15** was prepared by addition of mesyl chloride (320 L, 1.5 eq.) and triethylamine (770 L, 2 eq.) to a solution of (*E*)-*tert*-butyl 6-(3-hydroxyprop-1-en-1-yl)-1*H*-indole-1-carboxylate (800 mg, 2.9 mmol) in dichloromethane (30 mL). The mixture was stirred at room temperature for 1 h. It was then washed with water and brine, dried over Na_2_SO_4_, and concentrated to give crude **15**, which was used for the next step without further purification.

### 3.6. (E)-N-(t-Butoxycarbonyl)-6-(3-azidoprop-1-en-1-yl)-1H-indole (**16**)


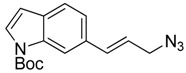


Crude (*E*)-*tert*-butyl 6-(3-((methylsulfonyl)oxy)prop-1-en-1-yl)-1*H*-indole-1-carboxylate (**15**) was dissolved in DMSO (10 mL). Sodium azide (355 mg, 5.46 mmol) was added at room temperature and the mixture was heated for 8 h at 80 °C. The mixture was cooled to room temperature and diluted with dichloromethane. It was then washed twice with water. The organic layer was dried over Na_2_SO_4_, and concentrated. The residue was purified by reversed phase preparative LC to give compound **16** (261 mg, 30%); ^1^H-NMR (300 MHz, CDCl_3_) (ppm): 8.25 (br s, 1H), 7.58 (d, *^4^J* = 3.6 Hz, 1H), 7.51 (d, *^3^J* = 8.1 Hz, 1H), 7.31 (dd, *^3^J* = 8.1 and *^4^J* = 1.5 Hz), 6.76 (d, *^3^J* = 15.9 Hz, 1H), 6.54 (d, *^4^J* = 3.6 Hz, 1H), 6.30 (dt, *^3^J* = 15.9 and *^3^J* = 6.6 Hz, 1H), 3.96 (d, *^3^J* = 6.6 Hz, 2H), 1.7 (s, 9H).

### 3.7. (E)-N-(2-Aminophenyl)-3-(1-cinnamyl-1H-1,2,3-triazol-4-yl)acrylamide (**click 1**)


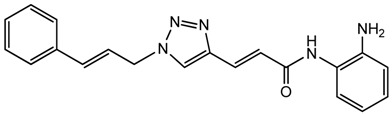


A mixture of cinnamyl azide **5** (0.18 g, 1.15 mmol), enyne intermediate **3** (0.27 g, 0.96 mmol), sodium ascorbate (38 mg, 0.192 mmol, 20 mol%) and CuSO_4_ (12 mg, 0.048 mmol, 5 mol%) in *t*-BuOH-H_2_O (2:1, 3.9 mL) was stirred at room temperature for 16 h. Water (20 mL) was added, and the product was extracted with ethyl acetate (30 mL, 3×). The combined organic layers were washed with brine (60 mL), dried with sodium sulfate, filtered, and concentrated. The resulting residue was subjected to column chromatography to yield the desired product (0.36 g, yield: 84%). The Boc-protected intermediate (0.36 g, 0.81 mmol) from the above reaction was dissolved in 20% TFA in dichloromethane (3.5 mL). The solution was stirred at room temperature for 1 h. The solvent was removed, and the reaction mixture was redissolved in 10 mL dichloromethane and concentrated again. The resulting residue was purified by column chromatography to give the desired product (0.25 g, yield: 90%).^1^H-NMR (500 MHz, CD_3_OD) (ppm): 8.31 (s, 2H), 7.76 (d, 1H, *^3^J* = 15.5 Hz), 7.26–7.46 (10H), 7.04 (d, 1H, *^3^J* = 16 Hz), 6.75 (d, 1H, *^3^J* = 15.5 Hz), 6.47 (dd, 1H, *^3^J* = 6.5 Hz, 16 Hz), 5.23 (dd, 2H, *^3^J* = 6.75 Hz and *^4^J* = 1.5 Hz); ^13^C-NMR (125 MHz, CD_3_OD) (ppm): 166.1, 143.7, 136.2, 135.6, 131.1, 128.7, 128.6, 128.4, 127.6, 126.8, 125.6, 125.0, 123.7, 122.2, 121.0, 52.4; ESI-TOF: Expected: 346.1662, Observed: 346.1661.

### 3.8. (E)-3-(1-((E)-3-(1H-Indol-6-yl)allyl)-1H-1,2,3-triazol-4-yl)-N-(2-aminophenyl)acrylamide (**click 2**)


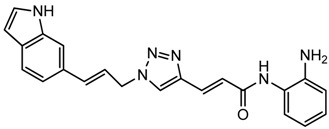


A mixture of (*E*)-*N*-(*t*-butoxycarbonyl)-6-(3-azidoprop-1-en-1-yl)-1*H*-indole (**16**, 0.125 g, 0.42 mmol), enyne intermediate **3** (0.100 g, 0.35 mmol), sodium ascorbate (14 mg, 0.071 mmol, 20 mol%) and CuSO_4_ (5 mg, 0.002 mmol, 5 mol%) in *t*-BuOH-H_2_O (2:1, 1.5 mL) was stirred at room temperature for 16 h. Water (10 mL) was added, and the product was extracted with ethyl acetate (15 mL, 3×). The combined organic layers were washed with brine (30 mL), dried with sodium sulfate, filtered, and concentrated. The resulting residue was subjected to column chromatography to yield the desired Boc-protected product (0.20 g, yield: 81%). The Boc-protected product (0.20 g, 0.34 mmol) from the above reaction was dissolved in 20% TFA in dichloromethane (2 mL) and the solution was stirred at room temperature for 1 h. The solvents were removed, and the reaction product was redissolved in 10 mL dichloromethane and concentrated again. The residue was purified by column chromatography to give click-2 (0.12 g, yield: 92%); ^1^H-NMR (500 MHz, CD_3_OD) (ppm): 8.30 (1H, s), 8.04 (1H, s), 7.71 (1H, d, *^3^J* = 15.5 Hz), 7.57 (1H, d, *^3^J* = 8.0 Hz), 7.51 (1H, s), 7.32 (1H, d, *^4^J*= 3.0 Hz), 7.27 (2H, td, *^3^J*= 8.5 and *^4^J* = 1.5 Hz), 7.06–7.13 (2H), 6.90–6.95 (2H), 6.79–6.83 (1H), 6.44–6.50 (2H), 5.27 (2H, dd, *^3^J*= 4.0 and *^4^J* = 1.5 Hz); ^13^C-NMR (125 MHz, CD_3_OD) (ppm): 165.5, 144.0, 142.1, 137.4, 136.8, 129.6, 129.4, 128.9, 127.3, 125.9, 125.8, 124.5, 124.0, 122.3, 120.4, 119.2, 118.5, 117.9, 117.6, 110.3, 101.5, 52.7; ESI-TOF: Expected: 385.1771, Observed: 385.1781.

### 3.9. (E)-3-(1-(2-((1H-Indol-6-yl)oxy)ethyl)-1H-1,2,3-triazol-4-yl)-N-(2-aminophenyl)acrylamide (**click 3**)


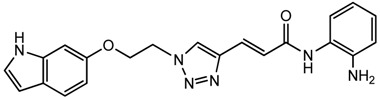


A mixture of 6-(2-azidoethoxy)-1 *H*-indole (**8**, 0.085 g, 0.42 mmol), enyne intermediate **3** (0.100 g, 0.35 mmol), sodium ascorbate (14 mg, 0.071 mmol, 20 mol%) and CuSO_4_ (5 mg, 0.002 mmol, 5 mol%) in *t*-BuOH-H_2_O (2:1, 1.5 mL) was stirred at room temperature for 16 h. Water (10 mL) was added, and the product was extracted with ethyl acetate (15 mL, 3×). The combined organic layers were washed with brine (30 mL), dried with sodium sulfate, filtered, and concentrated, and the resulting residue was subjected to column chromatography to yield the desired product (0.13 g, yield: 78%). The bis-Boc protected product (0.16 g, 0.33 mmol) from the above reaction was dissolved in 2 mL 20% TFA in dichloromethane and the mixture was stirred at room temperature for 1 h. Solvent was removed, and the reaction product was redissolved in 10 mL dichloromethane and concentrated again. The resulting residue was purified by column chromatography to give pure click-3 (0.12 g, yield: 95%). 1H-NMR (500 MHz, CD_3_OD) (ppm): 8.47 (1H, s), 8.37 (1H, s), 7.50-7.61 (5H), 7.42 (2H, d, *^4^J* = 1.5 Hz), 7.15-7.25 (5H), 7.029 (1H, d, *^3^J* = 15.5 Hz), 6.97 (1H, d, *^4^J* = 1.5 Hz), 6.65 (1H, dd, *^3^J* = 8.5 and *^4^J* = 2 Hz), 6.40 (1H, m), 4.83 (2H, t, *^3^J*= 4.5 Hz), 4.43 (2H, t, *^3^J* = 4.5 Hz); ^13^C-NMR (125 MHz, CD3OD) (ppm): 165.2, 154.5, 154.2, 143.6, 137.1, 130.1, 126.6, 125.3, 125.1, 123.3, 122.9, 121.6, 110.2, 101.9, 96.8, 80.8, 67.4, 50.4; ESI-TOF: Expected: 389.1720, Observed: 389.1714.

### 3.10. HDAC Enzyme Inhibition Assay

Serial dilutions of HDAC inhibitors were prepared in HDAC assay buffer (25 mM Tris/HCl, pH 8.0, 137 mM NaCl, 2.7 mM KCl, 1 mM MgCl_2_, pH 8) from DMSO stock solutions in 96-well assay plates (Fisher scientific, #07-200-309) and were pre-incubated for 2 h at ambient temperature in the presence of 125 μg/mL BSA and purified HDAC1 (#50051, BPS Bioscience, San Diego, CA, USA), HDAC2 (#50053, BPS Bioscience), or HDAC3/NcoR2 (#50003, BPS Bioscience) at concentrations of 1.25, 1.32, and 0.167 μg/mL, respectively. Following pre-incubation, Fluor-de-Lys^TM^ substrate (Enzo Life Sciences, Farmingdale, NY, USA) was added to a final concentration of 10 μM and plates were further incubated for 30 minutes at room temperature. The enzymatic reaction was stopped by addition of trichostatin A (Sigma-Aldrich, St. Louis, MO, USA, #T8552, final concentration: 100 nM) and trypsin (#02101179, MP Biomedicals, Solon, OH, USA) was added to reach a final concentration of 100 μg/mL. After a 15-minute incubation at room temperature, fluorescence was recorded using a Spectramax M2 fluorometer (Molecular Devices, Sunnyvale, CA, USA) with excitation at 365 nm and emission at 460 nm. IC_50_ values were calculated by using a sigmoidal dose-response (variable slope) equation in GraphPad Prism^®^ 5 for Windows (GraphPad Software, La Jolla, CA, USA).

### 3.11. In-Cell Deacetylase Inhibition Assay (DAC Assay)

GM15850 cells (an FRDA lymphoblastoid cell line from the Coriell Institute NIGMS collection) were seeded in 96-well plates at an appropriate density (100,000 cells/well) in 90 μL RPMI1640 medium containing 10% v/v fetal bovine serum (FBS), 1% v/v penicillin/streptomycin, and 1% v/v L-glutamine. Compound dilutions were made in 100% DMSO followed by parallel dilution in media with 2% DMSO. 10 μL of the compound dilutions were added to the cells to achieve the desired concentrations. The final concentration of DMSO in each well was 0.2%. Cells were incubated for 4 h at 37 °C with 5% CO_2_. After incubation, cells were centrifuged and the supernatant was removed. Cell pellets were washed with 100 μL phosphate-buffered saline (PBS) and then lysed with 45 μL lysis buffer (HDAC assay buffer at pH 8.0 (25 mM Tris/HCl, 137 mM NaCl, 2.7 mM KCl, 1 mM MgCl_2_ plus 1%, v/v, Igepal CA-630). To initiate the reaction, HDAC substrate KI-104 (Enzo Life Sciences) was added at a final concentration of 50 μM. The reaction was stopped after 30 min incubation by addition of 50 μL developer solution (6 mg/mL trypsin in HDAC assay buffer). The reaction was allowed to develop for 30 min at room temperature and the fluorescence signal was detected using a fluorometer (Spectramax M2, Molecular Devices, Sunnyvale, CA, USA) with excitation and emission wavelengths of 360 nm and 470 nm respectively. Data was fitted to a sigmoidal dose response equation with variable slope in GraphPad Prism 5.0 to determine IC_50_ values. Minimum and maximum values were fixed to the average fluorescence response of control wells with no cells and cells treated with 0.2% DMSO only respectively.

### 3.12. Brain Penetration Studies

Compound was dosed as a solution containing 5% DMSO by volume, 30% hydroxypropyl-β-cyclodextrin by weight in 100 mM acetate buffer at pH = 5.4. Dose volume was 1 mL/kg for iv dosing and 10 mL/kg for oral gavage (po dosing). Blood samples were taken at 0.083, 0.25, 0.5, 1, 2, 4, and 8 h post-dose. Serial sampling from the same animals (N = 3) was used for the p.o. dosed group. Terminal samples were obtained from animals dosed iv. Brain was collected at sacrifice to determine drug levels. Blood samples were collected in EDTA containing tubes and plasma was isolated. Plasma samples were analyzed by LC/MS/MS on an API4000 triple quadrupole (AB Sciex, Foster city, CA, USA) after protein precipitation from plasma or homogenized brain samples using acetonitrile containing an internal standard as the precipitant. Standards were prepared by serial dilution of analyte stock solution in corresponding matrix (rat plasma or brain tissue). Quantitation was generated automatically by the AB Sciex Analyst software. Data analysis was performed using Phoenix Winnonlin 6.1 (Pharsight Corporation, St. Louis, MO, USA) using the non-compartmental approach and the iv bolus or extravascular models provided in the software.

### 3.13. Acid Stability Determination

A 100 M solution of test compound was prepared by dilution of a 10 mM DMSO stock solution in a 0.01 M solution of HCl in deionized water. Immediately after mixing, an aliquot (100 μL) was sampled and analyzed by HPLC/UV. The area under the compound peak was determined and used as the time zero reference point. The remainder of the acid sample was incubated at 50 °C and samples were taken after 2, 4, and 24 h of incubation. These were analyzed by the same HPLC/UV method and the area of the peak corresponding to the test compound was measured. Percent remaining at a given time point was then calculated as the ratio of the area under the peak after incubation to that at time zero times 100.

## 4. Conclusions

In this report, we describe a new synthetic route to a class of benzamide HDAC inhibitors that show efficacy in restoring transcription to the silenced *FXN* gene in primary lymphocytes from Friedreich's ataxia patients. Our use of Cu(I)-catalyzed click chemistry enabled the facile synthesis of a series of derivatives of our current clinical candidate, HDACi **109**. Compound **109**, which has been shown to be effective in restoring *FXN* gene transcription both in primary patient cells and in animal models [8,9], suffers from two liabilities, namely, less than optimal brain penetration and poor stability under acidic conditions. Previous medicinal chemistry efforts (V.J. and J.R.R., unpublished) lead to the identification of two chemical features that can be incorporated into the pimelic o-aminobenzamide scaffold to overcome these limitations. These are replacement of the “left” amide with an ether, olefin or ketone to increase brain penetration and introduction of an unsaturated bond adjacent to the “right” amide to prevent formation of the benzimidazole product. Using click chemistry, we find that these two features can now be incorporated into a single molecule that improves on both properties while retaining the ability to increase *FXN* gene expression in patient cells. Importantly, compound click-1 has limited cytotoxicity issues compared to other benzamide HDAC inhibitors (such as MS-275), but retains its ability to inhibit recombinant HDACs (Table 1) and increase *FXN* gene expression in FRDA patient cells (Figure 3). These features suggest that this new class of compounds have improved efficacy as therapeutics for this neurodegenerative disease.

## Figures and Tables

* For consistency with previous publications [4,5,7,8] we use here the compound numbers employed in those previous reports.

**Table 1:** *HDAC* inhibition data for **109** and click compounds.

**ID**	**Structure**	**HDAC1 IC_50_(μM)**	**HDAC2 IC_50_(μM)**	**HDAC3 IC_50_(μM)**	**DAC^1^ IC_50_(μM)**
109	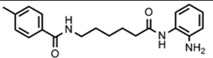	0.322	1.98	0.075	3.5
Click-1	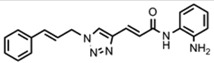	0.066	0.477	0.028	5.6
Click-2	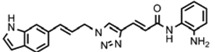	0.046	0.466	0.039	5.1
Click-3	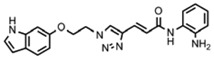	ND	ND	ND	29.4

1 In-cell deacetylase assay.
